# Host Genotype and Tissue Type Determine DWV Infection Intensity

**DOI:** 10.3389/finsc.2021.756690

**Published:** 2021-11-04

**Authors:** Hannah J. Penn, Michael Simone-Finstrom, Sarah Lang, Judy Chen, Kristen Healy

**Affiliations:** ^1^United States Department of Agriculture (USDA) Agricultural Research Service (ARS), Sugarcane Research Unit, Houma, LA, United States; ^2^United States Department of Agriculture (USDA) Agricultural Research Service (ARS), Honey Bee Breeding, Genetics and Physiology Research Laboratory, Baton Rouge, LA, United States; ^3^United States Department of Agriculture (USDA) Agricultural Research Service (ARS), Bee Research Laboratory, Beltsville, MD, United States; ^4^Department of Entomology, Louisiana State University Agriculture Center, Baton Rouge, LA, United States

**Keywords:** *Apis mellifera*, RNA virus, pathogenesis, virulence, genotype-by-genotype interactions, hypopharyngeal glands, Deformed wing virus

## Abstract

*Varroa* mite-vectored viruses such as Deformed wing virus (DWV) are of great concern for honey bee health as they can cause disease in individuals and increase colony mortality. Two genotypes of DWV (A and B) are prevalent in the United States and may have differential virulence and pathogenicity. Honey bee genetic stocks bred to resist *Varroa* mites also exhibit differential infection responses to the *Varroa* mite-vectored viruses. The goal of this project was to determine if interactions between host genotype could influence the overall infection levels and dissemination of DWV within honey bees. To do this, we injected DWV isolated from symptomatic adult bees into mite-free, newly emerged adult bees from five genetic stocks with varying levels of resistance to *Varroa* mites. We measured DWV-A and DWV-B dissemination among tissues chosen based on relevance to general health outcomes for 10 days. Injury from sham injections did not increase DWV-A levels but did increase DWV-B infections. DWV injection increased both DWV-A and DWV-B levels over time with significant host stock interactions. While we did not observe any differences in viral dissemination among host stocks, we found differences in virus genotype dissemination to different body parts. DWV-A exhibited the highest initial levels in heads and legs while the highest initial levels of DWV-B were found in heads and abdomens. These interactions underscore the need to evaluate viral genotype and tissue specificity in conjunction with host genotype, particularly when the host has been selected for traits relative to virus-vector and virus resistance.

## Introduction

Honey bees continue to maintain high rates of colony loss which has been attributed to multiple stressors, especially a variety of pests and pathogens. The ectoparasitic mite *Varroa destructor*, a common honey bee parasite worldwide, is considered to be a significant contributor to colony losses, in large part due to its propensity to vector multiple viruses [(1–3)]. Of the viruses that are vectored by *Varroa* mites, Deformed wing virus (DWV) is the most common and prevalent honey bee virus (4–6). The level of DWV infection at the colony level is typically positively correlated with the level of *Varroa* infestation (7–9). DWV, alone and in combination with *Varroa*, has often been associated with overwintering colony losses (10–13). The deadly association between DWV and *Varroa* mites has significantly altered the viral landscape and resulted in the collapse of millions of bee colonies worldwide (14).

DWV is currently recognized as a complex of three genotypes—A, B, and C (14–16). Of these genotypes, DWV-A was the originally dominant genotype prior to the invasion of *Varroa* mites and has been the most prevalent in the United States (US) (17). However, genotype DWV-B (or *Varroa* destructor virus 1 or VDV-1) is the most prevalent genotype in Europe and has been increasing in prevalence in the US in recent years (6, 13, 18). These genotypes are capable of cohabitation and recombination into possibly more virulent recombinants within the same host (19, 20). Infection by the different genotypes does not appear to cause different symptomologies at the individual level (21–23), but there have been debates on if one particular genotype is more virulent than another at the colony level (6, 9, 15, 16, 24–26). Recent literature has revealed competition can also exist between the two genotypes when cohabiting within the same host, resulting in lower pupal mortality and higher DWV-B loads relative to DWV-A (6, 26). Such varying and complex differences in results highlight the importance and necessity of investigating how host genotype, a potentially confounding factor in these studies, contributes to the infectivity and pathogenicity of different viral genotypes and ultimately impacts honey bee health at individual and colony levels.

Additionally, an enhanced understanding of DWV dissemination within the body of honey bees after infection would offer critical insight into transmissibility and symptom onset. DWV has been found throughout different tissues, and specific tissues have been identified as sites of replication for the virus and sites of infection that potentially lead to dissemination (27). Recent research found that the presence of DWV in the brains of honey bees could lead to changes in gene expression associated with behavioral maturation and foraging behavior (28). While there are differences in pathogenesis as a result of how the virus is transmitted (vertically and/or horizontally by conspecifics, directly, or indirectly *via Varroa*) (3, 29), injection *via Varroa* parasitism allows DWV to directly enter the hemolymph of developing pupae or adult bees and then spread to different parts of the body through hemolymph circulation. By assessing DWV dissemination, with particular emphasis on the injection route that causes the majority of covert and overt symptomologies, we can develop a better understanding of DWV transmission, pathogenicity, and epidemiology.

Honey bees have demonstrated variability in susceptibility to pests and pathogens, which may influence DWV dissemination and overall infection rates. *Varroa* resistant honey bee stocks have been bred to display hygienic behavior against *Varroa* mites, reducing *Varroa* numbers and DWV levels within colonies (30–34). In addition to *Varroa* resistance, multiple honey bee stocks demonstrate virus tolerance which has led to reduced individual mortality (35–38). Assessing the susceptibility and progression of DWV among different honey bee stocks will give a better understanding of DWV epidemiology and enable us to better manage DWV infection in honey bees. To achieve our study goals, we injected DWV into newly emerged adult bees from five genetic stocks (Carniolan, Italian, Pol-Line, Russian, and Saskatraz) varying in their susceptibility to *Varroa* mite (39–41) and potentially to some mite-vectored viruses (35, 37). Whereas Italian and Carniolan bees, commonly used throughout the industry, have been bred for honey production and colony size, Russian, Pol-Line, and Saskatraz bees have been specifically bred for *Varroa* resistance (42–45). Using these bee stocks, we determined the viral titer of two DWV genotypes (A and B) in different tissue types (abdomens, heads, hypopharyngeal glands, and rear legs) over the first 10 days of viral infection. These specific tissues were chosen because the abdomen is the site of *Varroa* feeding and DWV-injection (46–48); legs have been good indicators of viral dissemination in other species and can be non-destructively sampled if needed (27, 49); the head has been an indicator of bee infections that cause overt effects (29, 50); and hypopharyngeal glands in the head are food-related organs and provide possible transmission by food trophallaxis and had yet to be directly tested (3, 29, 51).

## Materials and Methods

### Source Colonies

All colonies were started from 2 to 2.5 lb. “packages” made on 3 May 2018 from 10 previously established Italian colonies at the USDA Honey Bee Breeding, Genetics, and Physiology Research Laboratory, Baton Rouge, LA (30°22′56″N, 91°10′40″W). Naturally-mated queens (*n* = 3 per stock) from the five genetic stocks were sourced from the USDA Bee Lab [Pol-Line and Russian, Saelao et al. (41)], a Canadian collaborating breeder (Saskatraz), or purchased from commercial suppliers (Carniolan and Italian). To increase worker acceptance of new queens, all queens were placed into new colonies on 4 May 2018 using plastic cages with a candy plug blocking the entrance that the queen and workers would chew through over a period of days for final release (52). Colonies were checked for the presence of the queen and the number of brood frames; those with fewer than three frames of bees were supplemented with additional brood frames on 17 May 2018 to equalize starting populations. All colonies were maintained following standard management practices in three apiaries within 6 km of each other (with Carniolan, Italian, and Saskatraz sharing an apiary, while Pol-Line and Russian colonies were kept in two separate apiaries). Colonies were not sampled until after 6 weeks post brood supplementation to allow time for population turnover to reflect queen genetics.

### Viral Isolation

To obtain the DWV viral solution, 20 adult bees with DWV infection symptoms were collected and ground in liquid nitrogen to a fine powder, homogenized in 10 ml PBS, and centrifuged at 5,000 rpm for at 4°C for 20 min following established protocols (29, 53, 54). The resulting supernatant containing viruses was filtered through a 0.2-micron filter (milex-GS syringe filter unit #SLGS033SS, Millipore Sigma, Burlington, MA, USA) to remove small tissue debris, fungi, and bacteria. qPCR was conducted to test for the presence of non-target viruses (Acute Bee Paralysis Virus, Black Queen Cell Virus, Chronic Bee Paralysis Virus, Israeli Acute Paralysis Virus, Kashmir Bee Virus, and Lake Sinai Virus) using the methods described below (primers in Supplementary Table 1). Viral quantification for non-specific DWV was performed by absolute quantification using the Standard Curve Method. All methods were previously established based on standard protocols (27, 34). One sample stock solution for DWV (measured using non-specific DWV primers) was selected based on negative results for non-target viruses and used to create the injection stock solution. Stock solutions were diluted to 10^5^, a biologically relevant, sublethal functional titer level for adult bees (1).

### Viral Injection

From July to October 2018, frames with emerging adult bees were brought into the lab where emerging bees from each colony were immediately uncapped and removed from the frame. Bees were inspected for *Varroa* mites and those with mites on them or in their pupal cells were discarded to try to mitigate the impacts of prior feeding and DWV transmission. To simulate the vectoring of DWV through the feeding of *Varroa* mites in a standardized way, bees were injected with 3.0 μL of DWV inoculum (DWV treatment). Three microliters of 1X PBS injection (PBS treatment) and no injection (included as bees may have had naturally occurring DWV infections, hereafter referred to as “control”) were implemented as controls. To reduce movement during injection, bees (including controls) were placed on ice for 2 min then injected (PBS and DWV treatments) using an UltraMicroPump with a SYS-Micro4 Controller (World Precision Instruments, Sarasota, FL, USA) with an infusion flow rate of 0.1 μL/s, following manufacturer's parameters. For the injection, a 30G needle (Hamilton Company, Reno, NV, USA) was inserted into the lateral abdomen between the fourth and fifth tergites, based on established protocols (54, 55). Following injections, bees were housed in cages (maximum of *n* = 30 bees) of the same treatment and colony according to standard methods (56). All cages were provided 50% sucrose solution and pollen substitute to ensure hypopharyngeal gland development; food was replenished as needed or when desiccated (56, 57). Cages were maintained in an incubator at 34°C and 85% relative humidity. A subset of three bees from each of the three treatments and 15 colonies were sacrificed at 1, 2-, 4-, 7-, and 10-days post-injection for a total of 675 bees and stored in sterile 1.5 mL centrifuge tube at −80°C. Given inherent mortality differences, more bees were treated as needed to obtain the necessarily time point samples.

### Dissection and RNA Isolation

To determine virus movement within the body of honey bees over time, the three-bee subset was dissected from each colony/treatment/stock/time point combination. Dissections were conducted over dry ice with each bee dissected with a new, sterilized blade. For each bee, the body was separated into legs, head, and abdomen with other body segments removed. The head was then embedded into beeswax (replaced for each dissection) and the hypopharyngeal gland removed according to previously published methods (58). Dissected tissues were stored in separate sterilized tubes on dry ice during dissection and long-term at −80°C until RNA extraction.

RNA was extracted for a single rear leg, the head (sans hypopharyngeal gland), the hypopharyngeal gland, and the abdomen for each of the three bees representing each combination of colony/treatment/stock/time (*n* = 2,700 RNA extractions in total). To extract virus RNA from hypopharyngeal glands, samples were placed in 30 μL lysis buffer and 30 μL Maxwell Homogenization buffer and vortexed. For legs (cut into pieces), heads, and abdomens, samples were placed in 200 μL lysis buffer and 200 μL Maxwell homogenization buffer, manually ground with a pestle (Sigma-Aldrich, St. Louis, Missouri, USA), and vortexed. All samples were then incubated for 90 min at 4°C. After incubation, 320 μL Maxwell Homogenization buffer was added to hypopharyngeal gland samples. Total RNA of each tissue sample was extracted using the Maxwell RSC 48 cartridges (Promega Corporation, Madison, Wisconsin, USA) according to standard procedures of Maxwell RSC simplyRNA tissue extraction kits and program (Promega Corporation, Madison, Wisconsin, USA). RNA was quantified *via* NanoDrop One (Thermo-Fisher Scientific Inc., Waltham, Massachusetts, USA) twice using 1 μL of sample each time. The mean ng/μL NanoDrop One readings were calculated per sample then used to determine the quantities of sample and nuclease-free water required to reach a sample concentration of 250 ng of RNA. RNA was stored in 0.6 mL elution tubes wrapped in parafilm (Bemis NA, Neenah, Wisconsin, USA) at −80°C until cDNA synthesis (59, 60).

### cDNA Synthesis and RT-qPCR

Frozen RNA samples were thawed on −20°C metal beads, briefly vortexed, then centrifuged. cDNA was then synthesized in two steps using Qiagen QuantiTect Reverse Transcription kits (Thermo-Fisher Scientific Inc., Waltham, Massachusetts, USA). For step one, 2 μL of gDNA wipeout solution was added to the mix of RNA and water for a total reaction volume of 14 μL per sample. Samples were incubated at 42°C for 2 min in a Bio-Rad T100 Thermal Cycler (Bio-Rad, Hercules, California, USA). Samples were briefly vortexed and centrifuged before the addition of 4 μL 5X Buffer, 1 μL of RT Primer mix, and 1 μL of RT enzyme per sample. Samples were again briefly vortexed and centrifuged then placed into the Bio-Rad T100 Thermal Cycler (42°C for 25 min then 95°C for 3 min). Synthesized cDNA was stored in strips tubes wrapped in parafilm at −80°C until RT-qPCR.

To quantify DWV-A and DWV-B levels (1, 22, 61, 62), each sample was replicated two times per primer pair for RT-qPCR analyses. All RT-qPCRs consisted of 5 μL SsoFast Universal SYBR Green supermix (Bio-Rad, Hercules, California, USA), 3 μL nuclease-free water, 0.5 μL forward primer, 0.5 μL reverse primer, and 1 μL cDNA from the sample. All reactions were run in Bio-Rad CFC 96 or Connect Thermal Cyclers (Bio-Rad, Hercules, California, USA) with all reactions of a specific primer occurring in the same machine. All samples were tested with DWV-A and DWV-B primers to determine infection levels (primers in Supplementary Table 1). The PCR cycling protocol for DWV-A was 95°C for 1 min followed by 40 cycles of 95°C for 10 s and 60°C for 15 s then 65°C for 5 s; while the protocol for DWV-B was 95°C for 5 min followed by 40 cycles of 95°C for 5 s and 52.5°C for 10 s then 72°C for 10 s. The thermal protocols included a melt-curve dissociation analysis to confirm product size. DWV-A and DWV-B results were quantified using the Standard Curve Method using linearized plasmid constructs. Quantified virus titer levels were log-transformed for analyses.

### Statistical Analyses

**Figure 1:**
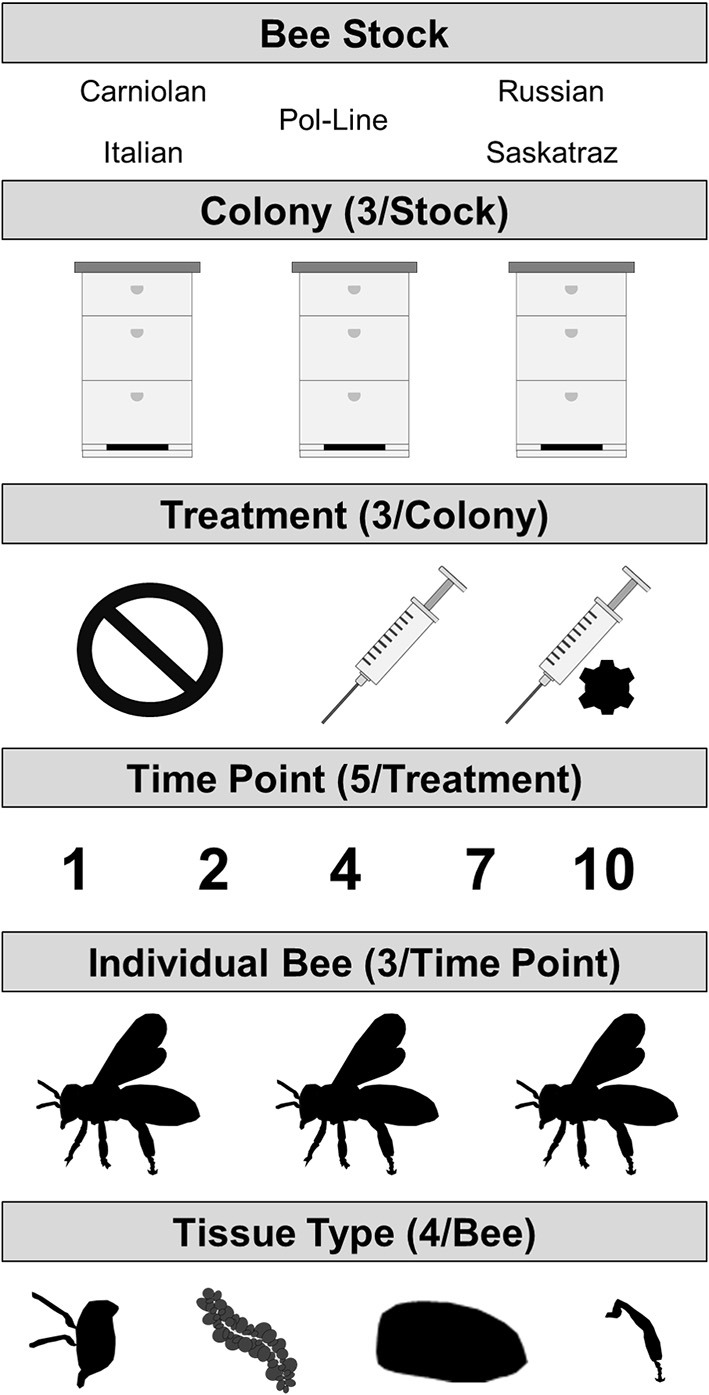
Experimental design where DWV types A and B were measured in four tissue types per bee which were collected from three individual bees for each combination of three treatments (no injection control/naturally occurring infection, PBS sham injection, or DWV injection) and five time points post treatment (days post treatment). This was repeated for three colonies of each of the five bee stocks.

We analyzed DWV levels in four tissue types per bee for three individual bees per timepoint (*n* = 5) and treatment (*n* = 3). This was replicated simultaneously for three colonies for each of five honey bee stocks for a total of 2,700 RNA extractions and 675 individual bees (Figure 1). The factors influencing levels of DWV-A and DWV-B were conducted using two general linear mixed models (one per virus genotype) or GLMMs (lme4 package) in R v 3.6.1 (63, 64). For all models, colony and bee ID were used as random effects to account for variation among colonies and individual bees (for tissue type) that were unaccounted for by stock. The following variables were considered for the fixed effects: treatment (no injection control, PBS injection, and DWV injection), tissue type (abdomen, head, hypopharyngeal gland, and legs), genetic stock (Carniolan, Italian, Pol-Line, Russian, and Saskatraz), time since treatment (1, 2, 4, 7, and 10 days), Log DWV (genotype B for the genotype A model and vice versa), all double and triple interaction effects of the previous variables, and a quadratic time term. The no injection control treatment (given that there was naturally occurring DWV infection present), the abdominal tissue (site of *Varroa* feeding), and the Italian bee stock (commercial standard) were specified as the intercept values; model results indicated in tables are relative to these values. Model fit was evaluated using estimated AICc and BIC scores using the ANOVA function in lmer; those with significantly lower scores (ΔAICc > 4) were used. When the scores were not different, the model containing more variables was used for ease of comparison between DWV-A and DWV-B models. *P*-values were estimated for all models using Satterthwaite's method in the lmerTest package (65). *Post-hoc* Tukey tests with a Sidak correction were conducted using emmeans package (66). All graphs were made using ggplot2 (67).

## Results

### Progression of Virus Genotypes

**Figure 2:**
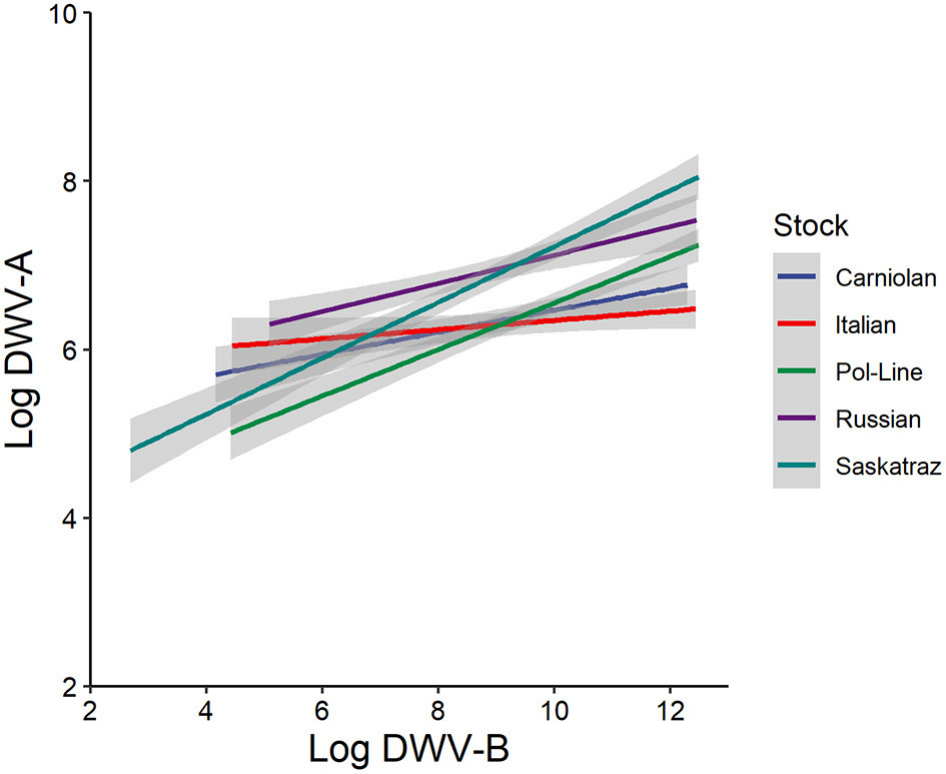
Log DWV-A titers as a function of log DWV-B titers relative to bee stock (*n* = 135 bees/stock) with all times, tissue types, and treatments pooled. Gray areas represent the standard error for each line. For individual and colony variation (see Supplementary Figure 1).

In both virus genotype models, the level of the alternate virus genotype was significant. Note, strains were not further characterized and these data are based on the primers (Supplementary Table 1). In the DWV-A model, both the model (Table 1) and fixed effects (Supplementary Table 2) indicated that greater levels of DWV-B were positively associated with an increase in DWV-A levels (Figure 2). The same occurred in the DWV-B model when DWV-A was included. In general (all treatments and tissue types combined), DWV-B levels were higher than DWV-A (ANOVA, DF = 1, 5,230, *F* = 1,125.482, and *p* < 0.001). Furthermore, DWV-B levels were higher than DWV-A when split out by day 1 (ANOVA, DF = 1, 1,037, *F* = 105.883, and *p* < 0.001) and day 10 (ANOVA, DF = 1, 1,064, *F* = 246.818, and *p* < 0.001). The higher DWV-B levels compared to DWV-A for all treatments are potentially due to higher levels of naturally occurring DWV-B infections. Bees (based on control treatments) started with naturally occurring DWV infections with an average of 10^6.08^ for DWV-A and 10^6.92^ for DWV-B, which continued to develop throughout the observation period.

**Table 1:** ANOVA summaries for DWV-A and DWV-B general linear mixed models.

	**Log DWV-A**						**Log DWV-B**					
**Variable**	**Sum Sq**	**Mean Sq**	**NumDF**	**DenDF**	***F*** **-value**	***P*** **-value**	**Sum Sq**	**Mean Sq**	**NumDF**	**DenDF**	***F*** **-value**	***P*** **-value**
Treatment	23.24	11.62	2	605.95	14.94	<0.001	12.59	6.293	2	641.62	16.34	<0.001
Tissue type	396.19	132.06	3	1,900.45	169.77	<0.001	386.81	128.935	3	1,876.53	334.77	<0.001
Stock	4.43	1.11	4	12.85	1.42	0.28	0.74	0.186	4	12.62	0.48	0.75
Log DWV-A							204.44	204.44	1	2,271.56	530.82	<0.001
Log DWV-B	339.13	339.13	1	2,417.48	435.95	<0.001						
Time	0.94	0.94	1	697.18	1.21	0.27	73.75	73.746	1	641.51	191.48	<0.001
Time^2^	1.40	1.40	1	662.89	1.80	0.18	52.35	52.354	1	636.6	135.93	<0.001
Treatment*Stock	16.65	2.08	8	591.16	2.68	0.01	2.95	0.369	8	632.71	0.96	0.47
Treatment* Tissue	28.62	4.77	6	1,817.96	6.13	0.00	5.28	0.88	6	1,859.27	2.28	0.03
Tissue *Stock	10.43	0.87	12	1,817.26	1.12	0.34	6.29	0.524	12	1,858.18	1.36	0.18
Treatment*Time	3.94	1.97	2	588.76	2.53	0.08	0.02	0.009	2	631.55	0.02	0.98
Tissue *Time	192.17	64.06	3	1,831.26	82.34	<0.001	80.19	26.731	3	1,866.7	69.40	<0.001
Stock*Time	0.83	0.21	4	590.39	0.27	0.90	4.68	1.171	4	631.05	3.04	0.02
Treatment*Stock*Time	13.59	1.7	8	588.79	2.18	0.03	1.73	0.216	8	631.3	0.56	0.81

*A total of 675 adult bees presenting the five stocks (135 bees/stock), three treatments (n = 45 bees/stock/treatment), and five timepoints (n = 9 bees/stock/treatment/time) were used in this analysis. These bees were dissected into four tissue types which were used for isolation of DWV titers (n = 2,700 RNA extractions total)*.

There was a strong treatment effect for both DWV-A and DWV-B (Table 1). Of the treatments, only the DWV injection treatment was significantly related to DWV-A levels in the fixed effects (Supplementary Table 2). Control and PBS treatments were similar to each other, and both had significantly lower DWV-A levels than the DWV treatment due to naturally occurring DWV infections (Table 2). There was a slightly different pattern for the DWV-B model—both PBS and DWV injection treatments were significant predictors of DWV-B copy number (Supplementary Table 2). Using Tukey comparisons, PBS had significantly greater levels of DWV-B than did the control but still lower levels than the DWV treatment (Table 2).

**Table 2:** Log-transformed mean virus levels combined for all time points combined (mean) and time of highest virus titers (peak day) for both DWV-A and DWV-B with the standard errors (SE).

**Type**	**Variable**	**Log DWV-A**				**Log DWV-B**			
		**Mean (SE)**	**Tukey**	**Peak day (SE)**	**Tukey**	**Mean (SE)**	**Tukey**	**Peak day (SE)**	**Tukey**
Treatment (*n* = 225 bees/ treatment)	Control	6.90 ± 0.23	A	5.85 ± 0.58	A	8.73 ± 0.23	A	6.10 ± 0.43	A
	PBS	6.87 ± 0.23	A	7.85 ± 0.58	B	9.47 ± 0.23	B	7.00 ± 0.43	A
	DWV	8.37 ± 0.23	B	6.80 ± 0.58	AB	10.05 ± 0.23	C	6.85 ± 0.43	A
Tissue (*n* = 675 bees/tissue type)	Abdomen	6.81 ± 0.22	A	7.07 ± 0.67	A	9.91 ± 0.22	A	6.60 ± 0.46	AB
	Hypo. Gland	7.39 ± 0.22	B	7.00 ± 0.67	A	9.06 ± 0.22	B	6.00 ± 0.46	A
	Head	7.44 ± 0.22	B	7.80 ± 0.67	A	9.86 ± 0.22	A	6.00 ± 0.46	A
	Leg	7.88 ± 0.22	C	5.47 ± 0.67	A	8.85 ± 0.22	C	8.00 ± 0.46	B
Stock (*n* = 135 bees/stock)	Italian	7.13 ± 0.49	A	7.75 ± 0.76	A	9.62 ± 0.49	A	5.50 ± 0.50	A
	Russian	8.21 ± 0.49	A	5.58 ± 0.76	A	8.84 ± 0.49	A	5.75 ± 0.50	A
	Pol-Line	6.59 ± 0.49	A	7.58 ± 0.76	A	10.03 ± 0.49	A	6.50 ± 0.50	AB
	Carniolan	7.20 ± 0.49	A	6.92 ± 0.76	A	9.58 ± 0.49	A	7.75 ± 0.50	B
	Saskatraz	7.77 ± 0.49	A	6.33 ± 0.76	A	9.01 ± 0.49	A	7.75 ± 0.50	B

“*Tukey” letters denote significant differences within each type using post-hoc Tukey tests (α = 0.05). “Type” sections were analyzed independently from each other as were the two DWV types. For “Treatment,” all tissue types and bee stocks were pooled; for “Tissue,” all treatments and stocks were pooled; and for “Stock” all treatments and tissue types were pooled for analyses*.

In the DWV-A model, neither the time nor the quadratic time term was statistically significant without interaction terms (in the corresponding sections below) in explaining levels of DWV-A (Tables 1, 2). However, there was a significant positive relationship of time (and negative with the quadratic term) to DWV-B levels (Table 1; Supplementary Table 2). There was only marginal evidence for a treatment by time interaction in the DWV-A model (Table 1). In the DWV-A model fixed effects, neither PBS nor DWV treatments were significant (Supplementary Table 2). Nor we did not find any evidence, even marginal, for a treatment by time interaction in the DWV-B model (Table 1; Supplementary Table 2). However, virus levels varied with other variables and interaction combinations over time (in the corresponding sections below).

### Virus Dissemination

**Figure 3:**
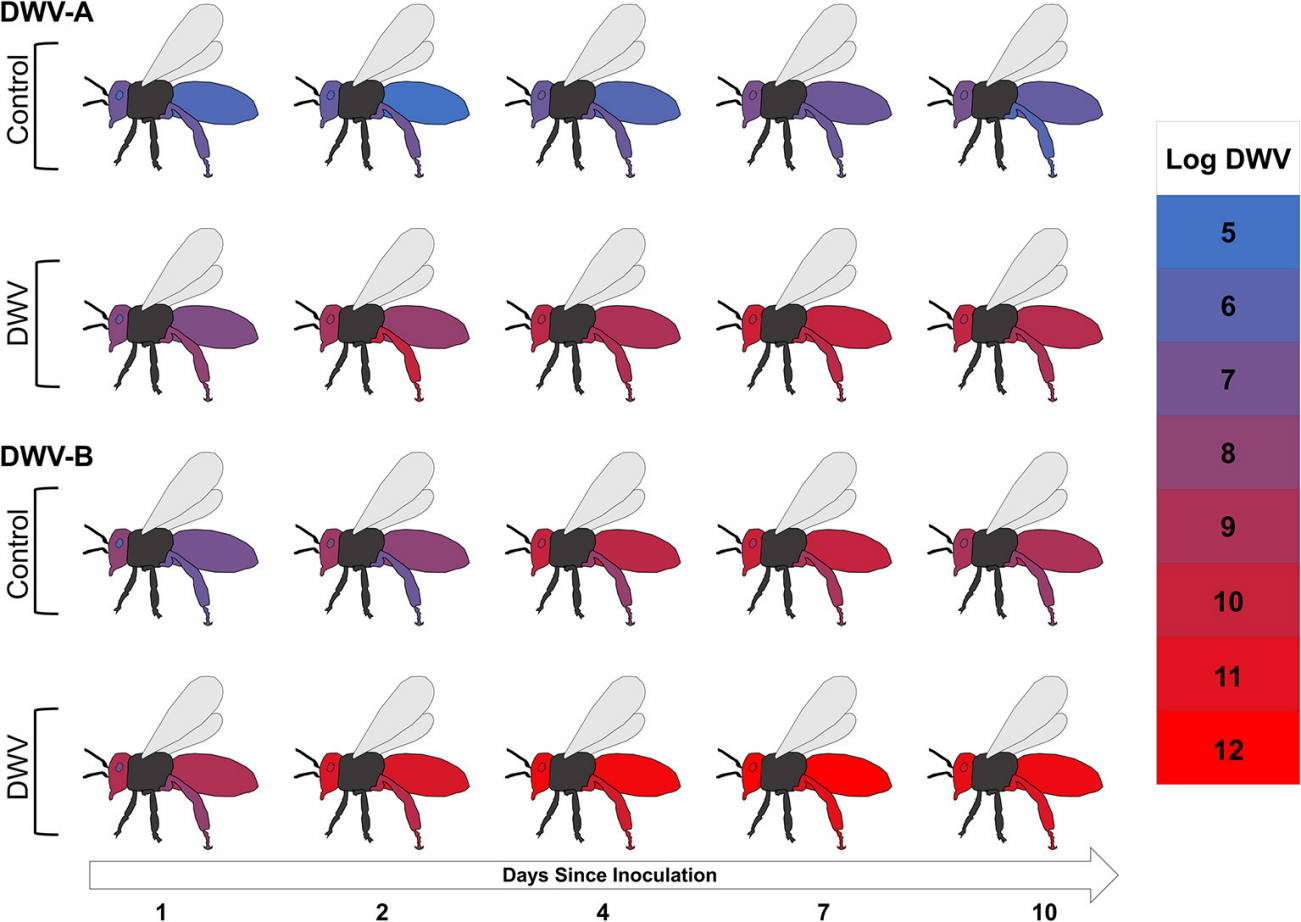
The mean Log DWV-A and DWV-B levels (color scale) 1, 2, 4, 7, and 10 days after inoculation for the four tissue types (head, hypopharyngeal gland, rear leg, and abdomen) for control and DWV-injection treatments with stocks pooled. Gray and black areas were not tested for either virus genotype. Information for the PBS-injection treatment can be found in Supplementary Figure 2.

When we looked at overall DWV-A and DWV-B viral levels (controlling for treatment, stock, and time), the tissue type variable was significant (Figure 3; Supplementary Figure 2; Table 1), with all tissue types having significant fixed effects (Supplementary Table 2). However, we saw opposite interactions between the two virus genotypes. In the DWV-A model, legs had the greatest virus levels while abdomens had the lowest levels among all examined tissues (Table 2). Alternatively, for DWV-B, abdomens had the greatest level while legs had the least (Table 2). For each virus genotype, head tissue consistently had relatively high levels of the virus compared to other tissues, making it potentially useful as an indicator of both DWV virus genotypes.

When tissue type by treatment interactions were analyzed, we found significant relationships for both virus models (Figure 3; Table 1), primarily due to the DWV treatment (Supplementary Table 2). The DWV treatment had higher levels of DWV-A than did PBS and control treatments for each tissue type (*post-hoc* tests not in Table 2). A similar pattern occurred for DWV-B; however, tissue types with PBS treatments had greater virus levels than did their associated control treatments (*post-hoc* tests not in Table 2).

We did not find any tissue type by bee stock interactions for either DWV-A or DWV-B models (Table 1; Supplementary Table 2) though there was a significant interaction of tissue type with time (Figure 3; Table 1). For the DWV-A model, legs were driving this trend (Supplementary Table 2), with the ending virus levels (day 10) being even lower than the starting levels (day 1). There was also an earlier (though not significantly so) peak at day 4 (Table 2). For the DWV-B model, all tissue types were significant (Supplementary Table 2), with legs having a significantly later peak than hypopharyngeal glands and heads (Table 2). We did not see any significant triple interactions for either virus using tissue type in combination with treatment or time in relation to stock; this makes sense given the lack of an initial interaction of stock and tissue type.

### Host Genotype Differences

**Figure 4:**
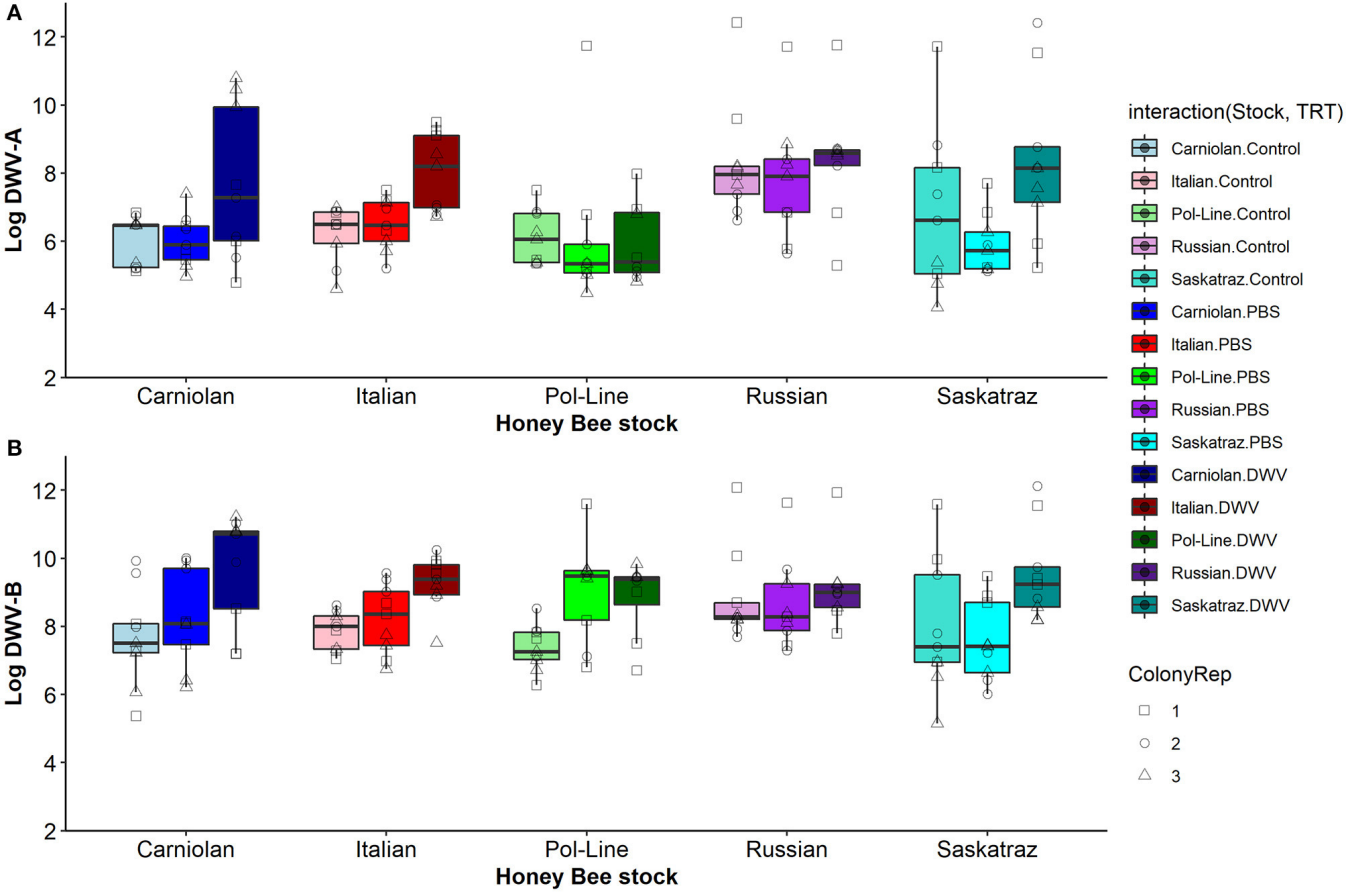
DWV levels in bee heads at time point 1 for **(A)** Log DWV-A and **(B)** DWV-B for the five genetic stocks and the three treatment groups (non-manipulated control bees, PBS injected bees, and DWV injected bees). Boxplots are in the style of Tukey where the box limits represent the lower 25% quantile and upper 75% quantile with the line representing the median. Individual points indicate individual bees tested with shapes indicate the colony replicate within each stock (indicated by color) and treatment (indicated by shade).

When we compared genetic stocks using only the control treatment (pooled across time), we found no differences in DWV-A levels (ANOVA, DF = 4, 883, *F* = 1.961, and *p* = 0.098), though numerically, Russian bees had the highest DWV-A levels (6.589 ± 0.105 log-transformed functional titer level) while Pol-Line (6.205 ± 0.105) had the lowest levels. When we separated the control treatment bees further by time, we found stock differences in DWV-A levels only for day 1 (ANOVA, DF = 4, 178, *F* = 10.645, and *p* < 0.001) but not for any other time point (ANOVA, Day 2: DF = 4, 171, *F* = 1.189, and *p* = 0.318; Day 4: DF = 4, 175, *F* = 1.040, and *p* = 0.388; Day 7: DF = 4, 178, *F* = 2.024, and *p* = 0.093; Day 10: DF = 4, 177, *F* = 1.756, and *p* = 0.140). On day 1, Russian bees had significantly higher DWV-A levels than all other stocks (Figure 4; Tukey HSD, α = 0.05). When we analyzed DWV-B levels in control bees for all times combined, we found significant differences among stocks (ANOVA, DF = 4, 855, *F* = 15.236, and *p* < 0.001). Using Tukey HSD comparisons combining all times (α = 0.05, letters denote significant differences), Russian (7.864 ± 0.156 a) and Saskatraz bees (7.677 ± 0.155 a) had significantly lower DWV-B levels than Carniolan (8.704 ± 00.157 b), Italian (8.717 ± 0.155 b), and Pol-Line bees (9.085 ± 0.156 b). When we separated the control treatment bees by time, we found stock differences in DWV-B levels for all days (Tukey HSD, α = 0.05). On day 1, Russian bees had higher DWV-B levels than Pol-Line and Saskatraz. However, by day 2, Pol-Line bees had the highest levels but were only significantly higher than Saskatraz. This trend further solidified on day 4, where Pol-Line and Italian bees had higher levels of DWV-B than Carniolan, Russian, and Saskatraz bees. Day 7 was very similar, but with Carniolan bees joining the ranks of Pol-Line and Italian. On the final day, day 10, Pol-Line had similar DWV-B levels to Carniolan but higher levels than all other stocks and Carniolan had higher levels than Russian bees.

Without considering interaction terms, when we grouped all treatments, the genetic stock of the bees had no impact on the levels of either DWV-A or DWV-B (Table 1). When looking at the fixed effects of the DWV-A model (Supplementary Table 2), Russian bees were marginally positively correlated with increased DWV-A virus levels. This trend does not hold for DWV-B. In general, the Russian stock tended to have higher levels of DWV-A while Pol-Line had the lowest with Italian, Carniolan, and Saskatraz stocks middling (Table 2), though none were significantly different. The opposite (though again not statistically significant) occurred for DWV-B, with Pol-Line having the higher trending levels and Russian bees having the lowest. This suggests potential viral genotype by host genotype interactions between viral genotypes.

**Figure 5:**
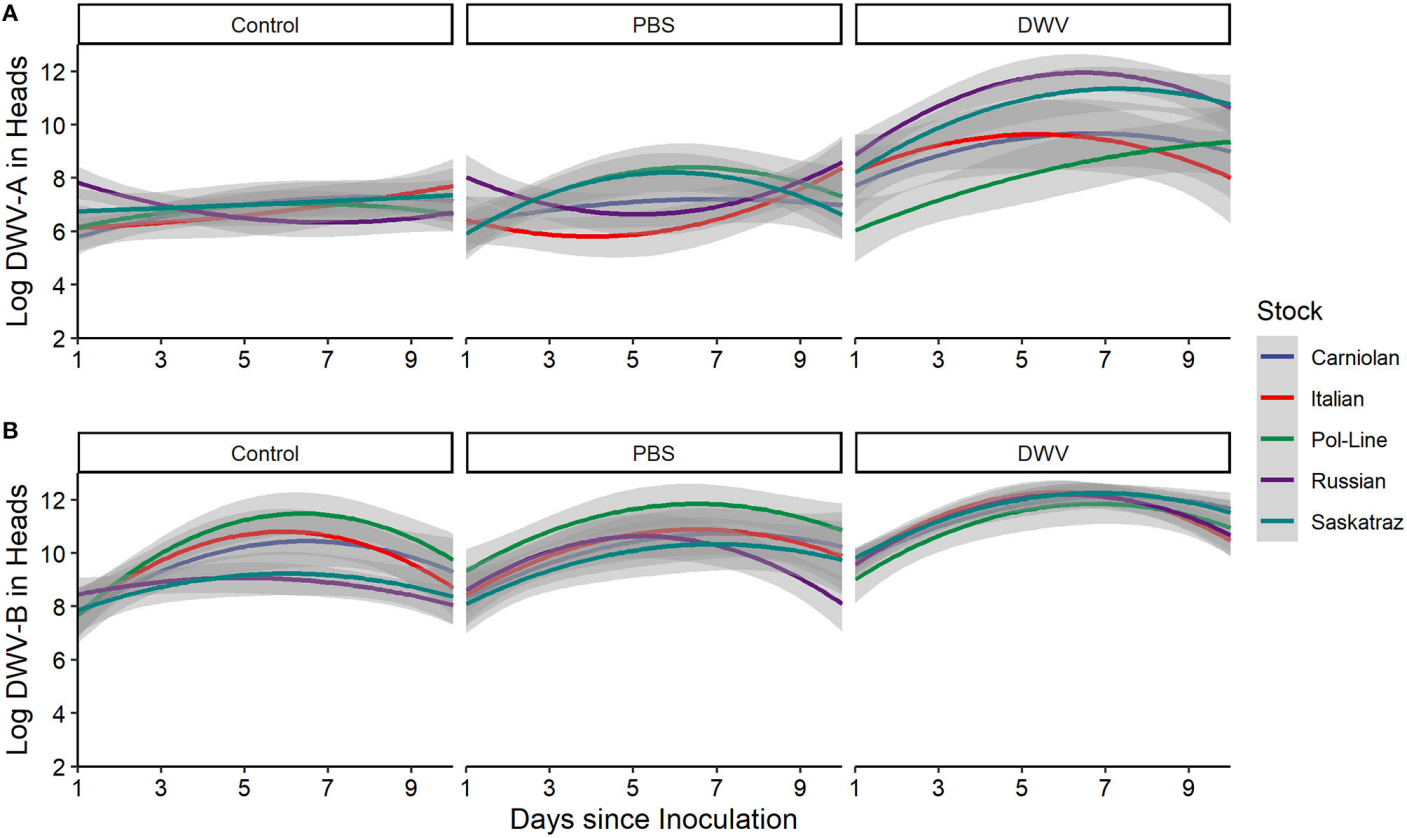
DWV levels in bee heads for five genetic stocks over time (1, 2, 4, 7, and 10 days after inoculation) for **(A)** Log DWV-A and **(B)** DWV-B levels. Gray areas represent the standard error for each line. Similar patterns were noted for the other tissue types (see Supplementary Figures 4–6). Additionally, data for PBS and DWV injections made relative to control treatments for the same colony and time point indicate similar trends (Supplementary Figure 3).

There was a treatment by stock interaction in DWV-A (Table 1), primarily driven in the fixed effects (Supplementary Table 2) by the Pol-Line and DWV treatment when compared to the intercept (Italian Control). However, we did not see any treatment by stock interactions in the DWV-B model (Figure 5). Even in the model fixed effects, no stock/treatment combinations were even marginally significant. For the stock by time interaction variable in the DWV-A model, the Russian stock exhibited significant fixed effects within the model (Supplementary Table 2) though the overall interaction variable was not significant (Table 1; Figure 5; Supplementary Figures 3–6). Alternatively, the DWV-B did have a significant stock by time interaction (Table 1) where DWV-B levels peaked at different times based on stocks (Table 2).

When we considered the stock, treatment, and time interactions, DWV-A had significant effects while DWV-B did not (Table 1). When we looked at the DWV-A model fixed effects, only stock interactions with the DWV treatment were significant over time (Supplementary Table 2), with all stocks but Carniolan being significant. When looking at Figure 5A, we saw few stock differences in DWV-A levels over time within the control treatment. In the PBS treatment, we observed that Pol-Line and Saskatraz appeared to increase over time with a positive peak; but Russian, Italian, and Carniolan stocks had a smaller increase or even a decrease in DWV-A over time. For DWV treatments, we observed that all stocks but Pol-Line peak and start decreasing in DWV-A over time. Pol-Line started on the lower end of DWV-A levels and increased linearly over time, with day 10 levels still being at the lower end of DWV-A levels. When we looked at the same three-way interaction term for DWV-B levels (Figure 5B), we did not see a significant relationship (Table 1), with no significant interactions in the fixed effects (Supplementary Table 2).

## Discussion

Despite the presence of naturally occurring DWV infection in the no injection and PBS controls, our DWV injection was effective—both DWV-A and DWV-B titers increased for the DWV injection. DWV-A levels did not change over the 10-day study period in either the PBS injection or the no manipulation control treatments. For DWV-B, we observed that injection alone, as indicated by the PBS injection, increased DWV-B titers with a synergistic effect seen in the DWV injection. Such a reaction to PBS injections might indicate that damage from mite feeding in adults can be compounded when the bee has a previously existing DWV-B infection, particularly if the mite also vectors DWV. This is particularly concerning if DWV-A and DWV-B are routinely co-occurring (6). We also found that DWV-A and DWV-B levels were positively associated, similar to prior work in injected pupae (23). Prior work on injected pupae has found that these genotypes do compete (26), and the data in this experiment do not necessarily refute this given that either genotype might have replicated more so when not in the presence of the other. However, the extent of this association seemed to differ based on host genotype (Figure 2), suggesting possible interactions between host and viral genotypes.

In general, we found that DWV-B levels were greater than that of DWV-A and accumulated more quickly. While this could be in part due to the ~1-log fold higher natural starting infections of DWV-B, for both virus genotypes, we found similar titer levels at the peak of infection (10^10−12^) and systemic, multi-day infections (68). In terms of DWV dissemination within the adult body over time, we observed viral genotype but not host genotype interactions. For DWV-A, rear legs and heads had the highest initial levels while abdomens had the lowest. Rear legs had on average the highest levels of DWV-A for the entire experiment, but there were no differences among tissue types in peak time. For DWV-B, heads and abdomens started with the highest levels, while rear legs had the lowest levels. The heads and abdomens also had a significantly shorter time to peak DWV-B levels than did the rear legs. The viral titers in hypopharyngeal glands were intermediate for both virus genotypes throughout the experiment, indicating a potential route of the virus transmission to larva from nurse bees along with worker-worker and worker-queen transmission (69). While legs are used in other insect systems to track virus titers over time (70), legs may not be entirely reliable for these two DWV genotypes in honey bees. Given the PBS injection data, the removal of the leg may induce virus replication, skewing later samples (22, 71). Differences between A and B maybe due in part to recombination of the two types (19, 26), so need to be more precisely evaluated in further studies.

The five tested honey bee genetic stocks differed in their levels of DWV-A and DWV-B and how infection levels varied over time. Overall, Russian bees had the highest levels of both virus genotypes in the control treatment heads, though only DWV-A was significant. After 1 day of treatment, Russian bees in the control treatment heads had higher levels of both DWV-A compared to all other stocks and higher levels of DWV-B compared to Pol-Line and Saskatraz bees, indicating potential baseline differences in virus levels that may have influenced stock interaction with the PBS and DWV treatments. Additionally, we observed bee stock differences in DWV-A and DWV-B in response to PBS and DWV injections, indicating baseline differences in how genetic stocks react to physical damage in addition to differences in potential virus resistance. For DWV-A levels, we observed few differences among stocks over time in the control treatment; but PBS and DWV injection treatments altered DWV-A levels over time based on stock. In PBS injections, Italian and Russian bees were similar (concave pattern over time), Pol-Line and Saskatraz were similar (convex pattern over time), and Carniolan bees were in between the two groups (a straight line over time, Figure 5). In the DWV injection, all stocks but Pol-Line exhibited convex patterns over time, while Pol-Line started at a lower level than all other stocks and linearly increased over time. Results were different for DWV-B levels, which only showed differences among bee stocks in the control and PBS treatments but not the DWV injection (Figures 4, 5).

How the bee stocks interact with injection treatment and virus genotype over time indicate that there are clusters of stocks but that these groupings differ based on the DWV genotype being evaluated. Saelao et al. (41) indicated several genetic clusters of bee stocks, with Pol-Line differentiated from Carniolan, Italian, and Russian stocks. These genetic clusters based on breeding practices for hygienic behavior or *Varroa*-mite suppression might also result in stock-related mite-vectored virus resistance (34, 72). Furthermore, host genotype by viral genotype interactions may help account for variation in the prevalence and distribution of DWV genotypes globally (6, 26, 73). The mechanisms for these bee genetic differences have not been well-studied but may involve the genetic differences in host physiological responses such as immune response and vitellogenin expression. For instance, vitellogenin and related physiological responses for the individual and the colony (74, 75) is differentially expressed by different bee genotypes (76, 77). Other bee stock-related differences, such as gut microbiota community structure, could also influence their ability to resist virus genotypes differentially (78–81).

Our results suggest that the interaction between genetic background of honey bee hosts and viral genotype may influence DWV infection levels over time. These data may indicate some tradeoff in resistance to different virus genotypes within the bee stocks bred for *Varroa* mite resistance. Additionally, we observed virus genotype differences in dissemination through the adult body. Future studies should focus on potential physiological differences in how viral genotypes operate interact with host immune responses. Such differences may be important for how the bees later cope physically or behaviorally with the virus infection and replication (82). We recommend that future work is necessary to evaluate bee host genotype by virus genotype interactions to better understand how breeding for *Varroa* mite resistance might confer virus resistance and influence virus genotype tradeoffs.

## Conclusion

Our study aimed to determine if five honey bee genotypes with differential levels of *Varroa* mite resistance also differed in dissemination patterns of two *Varroa* mite vectored DWV genotypes (A and B) in newly emerged adult bees. We found that titers of the two DWV genotypes were positively associated with each other and varied significantly but differently with our treatments. The two virus genotypes also significantly differed in dissemination location over time, while bee genotype did not impact dissemination trends. Aside from dissemination, overall infection levels were impacted by an interaction of bee and virus genotypes. This may indicate that while different breeding programs might have similar outcomes for *Varroa* mite resistance, mite-resistant bee stocks are not necessarily consistent in their interactions with mite-vectored viruses.

## Data Availability Statement

The raw data supporting the conclusions of this article will be made available by the authors, without undue reservation.

## Author Contributions

MS-F, KH, and JC designed the research. HP and SL performed the experiments and wrote the manuscript. HP conducted the statistics and analyzed the data. All authors edited the manuscript, contributed to this article, and approved the submitted version.

## Funding

This work was possible from USDA NIFA Grant 2017-69004-26515 and the USDA ARS, research plan 6050-21000-014-00D.

## Conflict of Interest

The authors declare that the research was conducted in the absence of any commercial or financial relationships that could be construed as a potential conflict of interest.

## Publisher's Note

All claims expressed in this article are solely those of the authors and do not necessarily represent those of their affiliated organizations, or those of the publisher, the editors and the reviewers. Any product that may be evaluated in this article, or claim that may be made by its manufacturer, is not guaranteed or endorsed by the publisher.
