# Supplementary material for: Host Genotype and Tissue Type Determine DWV Infection Intensity
Source: Front Insect Sci. 2021 Nov 4;1:756690. doi: 10.3389/finsc.2021.756690 (PMC10926404; doi:10.3389/finsc.2021.756690)

Supplementary Material

Supplementary Table 1. Primers used for virus detection and sample quality.

Primer Target	Forward Sequence	Reverse Sequence	Reference
AKI (ABPV, KBV, and IAPV combination)	ACCGACAAAGGGTATGATGC	CTTGAGTTTGCGGTGTTCCT	Roy et al. J Apic Sci DOI:10.2478/v10289-012-0014-x
BQCV	TTTAGAGCGAATTCGGAAACA	GGCGTACCGATAAAGATGGA	Boncristiani et al. 2012, J Ins Phys DOI:10.1016/j.jinsphys.2011.12.011
CBPV	CGCAAGTACGCCTTGATAAAGAAC	ACTACTAGAAACTCGTCGCTTCG	Blanchard et al. 2007, J Virol Meth DOI:10.1016/j.jviromet.2006.11.021
DWV, non-specific	GGACCATCCTTCCAGTCTACGAT	CTGTAGGTTGTGCTCCTGATGAAGA	Ryabov et al. 2014, Plos Pathogens DOI:10.1371/journal.ppat.1004230.s008
DWV-A	GAGATTGAAGCGCATGAACA	TGAATTCAGTGTCGCCCATA	Boncristiani et al. 2012, J Ins Phys DOI:10.1016/j.jinsphys.2011.12.011
DWV-B	CTGTAGTTAAGCGGTTATTAGAA	GGTGCTTCTGGAACAGCGGAA	Ryabov et al. 2014, Plos Pathogens DOI:10.1371/journal.ppat.1004230.s008
LSV	CGTGCGGACCTCATTTCTTCATGT	CTGCGAAGCACTAAAGCGTT	Daughenbaugh et al. 2015, Viruses DOI:10.3390/v7062772
β-actin	AGGAATGGAAGCTTGCGGTA	AATTTTCATGGTGGATGGTGC	Ryabov et al. 2014, Plos Pathogens DOI:10.1371/journal.ppat.1004230.s008

Supplementary Table 2. General linear mixed model fixed effects for Log DWV-A and Log DWV-B models. Control treatment, Italian stock, and abdomen dissection components were used in the model intercept.

		Log DWV-A					Log DWV-B				
Type	Variable	Estimate	Std. Error	df	*t*-value	*P*-value	Estimate	Std. Error	df	*t*-value	*P*-value
(Intercept)	(Intercept)	1.04	0.64	29.73	1.62	0.12	5.13	0.62	24.83	8.31	0.00
Treatment	PBS	-0.30	0.54	615.20	-0.56	0.57	0.31	0.52	645.70	0.60	0.55
	DWV	2.22	0.54	618.00	4.10	0.00	1.13	0.52	651.70	2.19	0.03
Tissue Type	Hyp. Glands (G)	0.99	0.15	1831.00	6.67	0.00	-0.96	0.10	1858.00	-9.26	<0.001
	Head (H)	0.77	0.15	1827.00	5.19	0.00	0.35	0.10	1864.00	3.37	0.00
	Leg (L)	2.27	0.16	1848.00	14.37	<0.001	-1.45	0.11	1884.00	-12.80	<0.001
Stock	Russian (Rus)	1.56	0.86	23.88	1.81	0.08	-0.78	0.85	22.30	-0.92	0.37
	Pol-Line (Pol)	0.24	0.86	23.88	0.28	0.78	-0.14	0.85	22.28	-0.17	0.87
	Carniolan (Car)	0.24	0.86	23.94	0.27	0.79	-0.29	0.85	22.31	-0.35	0.73
	Saskatrax (Sas)	0.92	0.86	23.96	1.07	0.30	-1.18	0.85	22.31	-1.39	0.18
Virus titers	Log DWV-A						0.31	0.01	2272.00	23.04	<0.001
	Log DWV-B	0.50	0.02	2417.00	20.88	<0.001					
Time	Time	0.01	0.10	658.20	0.11	0.91	0.96	0.10	644.00	10.01	<0.001
$Time^2$	$Time^2$	0.01	0.01	662.90	1.34	0.18	-0.08	0.01	636.60	-11.66	<0.001
Treatment*Stock	PBS*Rus	-0.75	0.76	590.40	-1.00	0.32	0.95	0.73	631.50	1.31	0.19
	DWV*Rus	-0.61	0.76	588.90	-0.80	0.42	0.22	0.73	631.00	0.30	0.76
	PBS*Pol	0.02	0.76	591.60	0.02	0.98	0.95	0.73	632.00	1.31	0.19
	DWV*Pol	-2.49	0.76	590.30	-3.29	0.00	0.03	0.73	634.80	0.04	0.97
	PBS*Car	0.67	0.76	592.80	0.88	0.38	-0.20	0.73	633.40	-0.27	0.79
	DWV*Car	-0.65	0.76	589.90	-0.86	0.39	0.19	0.73	631.60	0.27	0.79
	PBS*Sas	0.14	0.76	591.30	0.19	0.85	0.39	0.73	632.20	0.54	0.59
	DWV*Sas	-1.02	0.76	588.80	-1.35	0.18	0.77	0.73	631.00	1.07	0.29
Treatment* Tissue	PBS*G	-0.02	0.12	1812.00	-0.14	0.89	0.02	0.08	1856.00	0.27	0.79
	DWV*G	-0.61	0.12	1812.00	-5.15	0.00	0.03	0.08	1861.00	0.36	0.72
	PBS*H	0.01	0.12	1812.00	0.12	0.91	-0.12	0.08	1855.00	-1.46	0.15

	DWV*H	-0.24	0.12	1818.00	-2.00	0.05	-0.24	0.08	1857.00	-2.91	0.00
	PBS*L	0.15	0.13	1826.00	1.14	0.25	-0.01	0.09	1863.00	-0.11	0.91
	DWV*L	-0.22	0.13	1826.00	-1.76	0.08	-0.01	0.09	1864.00	-0.15	0.88
Dissection*Stock	G*Rus	-0.05	0.15	1809.00	-0.33	0.74	0.03	0.11	1854.00	0.25	0.80
	H*Rus	-0.01	0.15	1810.00	-0.05	0.96	0.09	0.11	1854.00	0.83	0.41
	L*Rus	-0.18	0.16	1824.00	-1.13	0.26	-0.07	0.11	1862.00	-0.62	0.53
	G*Pol	-0.16	0.15	1811.00	-1.06	0.29	-0.06	0.11	1855.00	-0.59	0.56
	H*Pol	0.09	0.15	1811.00	0.60	0.55	-0.08	0.11	1855.00	-0.77	0.44
	L*Pol	-0.18	0.16	1827.00	-1.11	0.27	-0.10	0.12	1863.00	-0.83	0.41
	G*Car	-0.11	0.15	1815.00	-0.73	0.47	-0.23	0.11	1856.00	-2.08	0.04
	H*Car	-0.09	0.15	1812.00	-0.61	0.55	-0.10	0.11	1855.00	-0.92	0.36
	L*Car	-0.18	0.17	1829.00	-1.07	0.29	-0.10	0.12	1864.00	-0.85	0.39
	G*Sas	-0.37	0.15	1811.00	-2.42	0.02	0.07	0.11	1856.00	0.64	0.52
	H*Sas	-0.03	0.15	1811.00	-0.19	0.85	0.08	0.11	1855.00	0.70	0.48
	L*Sas	-0.20	0.16	1826.00	-1.22	0.22	-0.12	0.11	1863.00	-1.08	0.28
Treatment*Time	PBS*Time	-0.02	0.09	588.80	-0.22	0.83	0.07	0.09	630.60	0.83	0.40
	DWV*Time	-0.14	0.09	587.70	-1.54	0.12	0.03	0.09	630.80	0.35	0.73
Dissection*Time	G*Time	-0.01	0.01	1814.00	-0.78	0.43	0.03	0.01	1855.00	2.53	0.01
	H*Time	-0.01	0.01	1828.00	-0.86	0.39	-0.06	0.01	1857.00	-5.61	0.00
	L*Time	-0.21	0.02	1827.00	-13.59	<0.001	0.10	0.01	1883.00	8.71	<0.001
Stock*Time	Rus*Time	-0.19	0.09	588.20	-2.08	0.04	-0.03	0.09	631.70	-0.35	0.73
	Pol*Time	-0.13	0.09	588.90	-1.39	0.17	0.15	0.09	630.50	1.65	0.10
	Car*Time	-0.06	0.09	591.50	-0.65	0.52	0.10	0.09	632.20	1.13	0.26
	Sas*Time	-0.07	0.09	588.00	-0.71	0.48	0.04	0.09	630.50	0.46	0.65
Treatment*Stock*Time	PBS*Rus*Time	0.25	0.13	587.60	1.96	0.05	-0.16	0.12	630.60	-1.25	0.21
	DWV*Rus*Time	0.34	0.13	587.70	2.66	0.01	0.00	0.12	632.20	0.03	0.98
	PBS*Pol*Time	0.06	0.13	588.50	0.47	0.64	-0.14	0.12	630.40	-1.09	0.28
	DWV*Pol*Time	0.39	0.13	588.50	2.98	0.00	-0.12	0.12	632.70	-0.95	0.34
	PBS*Car*Time	-0.01	0.13	591.40	-0.10	0.92	-0.04	0.12	632.40	-0.29	0.77
	DWV*Car*Time	0.15	0.13	589.20	1.14	0.26	-0.03	0.12	631.40	-0.28	0.78
	PBS*Sas*Time	0.01	0.13	588.10	0.10	0.92	0.00	0.12	630.40	-0.03	0.98
	DWV*Sas*Time	0.29	0.13	587.90	2.23	0.03	-0.01	0.12	631.70	-0.05	0.96

Supplementary Figure 1. Log DWV-A titers as a function of log DWV-B titers relative to bee stock (n = 135 bees/stock) with all times and treatments pooled. Gray areas represent the standard error for each line. Individual points indicate individual tissue RNA extractions (n = 2700 total) with shapes indicating source colonies (ColonyRep) and color indicating bee stock.

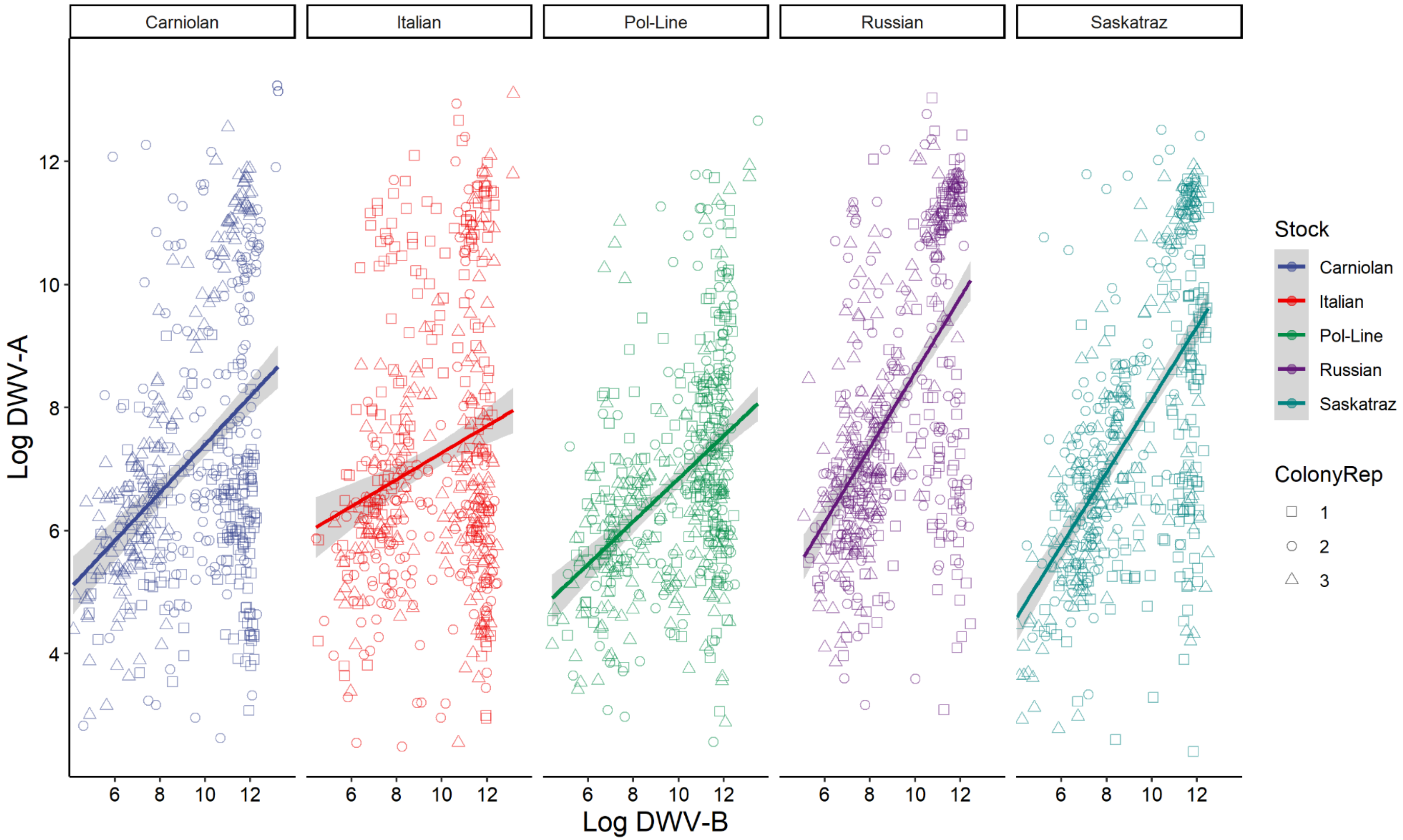

Supplementary Figure 2. *PBS injection* treatment mean Log DWV-A and DWV-B levels (color scales) 1, 2, 4, 7, and 10 days after inoculation for the four tissue types (head, hypopharyngeal gland, rear leg, and abdomen) with stocks pooled. Gray and black areas were not tested for either virus genotype.

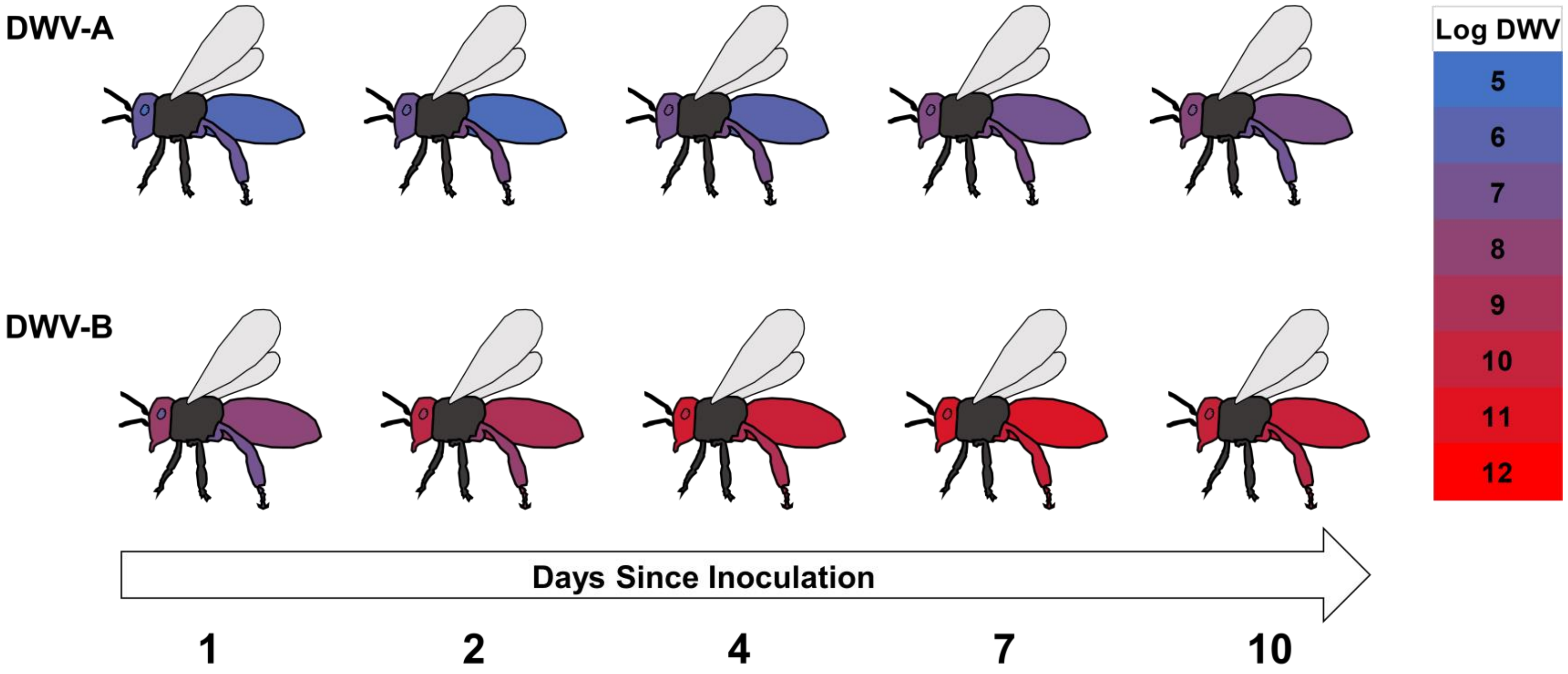

Supplementary Figure 3. DWV levels in *head* tissues 1, 2, 4, 7, and 10 days after inoculation for the five genetic stocks for (A) Log DWV-A and (B) DWV-B levels for *heads* only. Values for PBS and DWV treatments were made relative to control treatments (y = 0) for the same colony and time point. Gray areas represent the standard error for each line. Individual points indicate individual bees with shapes associated with colony replicate and color indicating stock. For standard view of these data, reference Figure 5 in the manuscript.

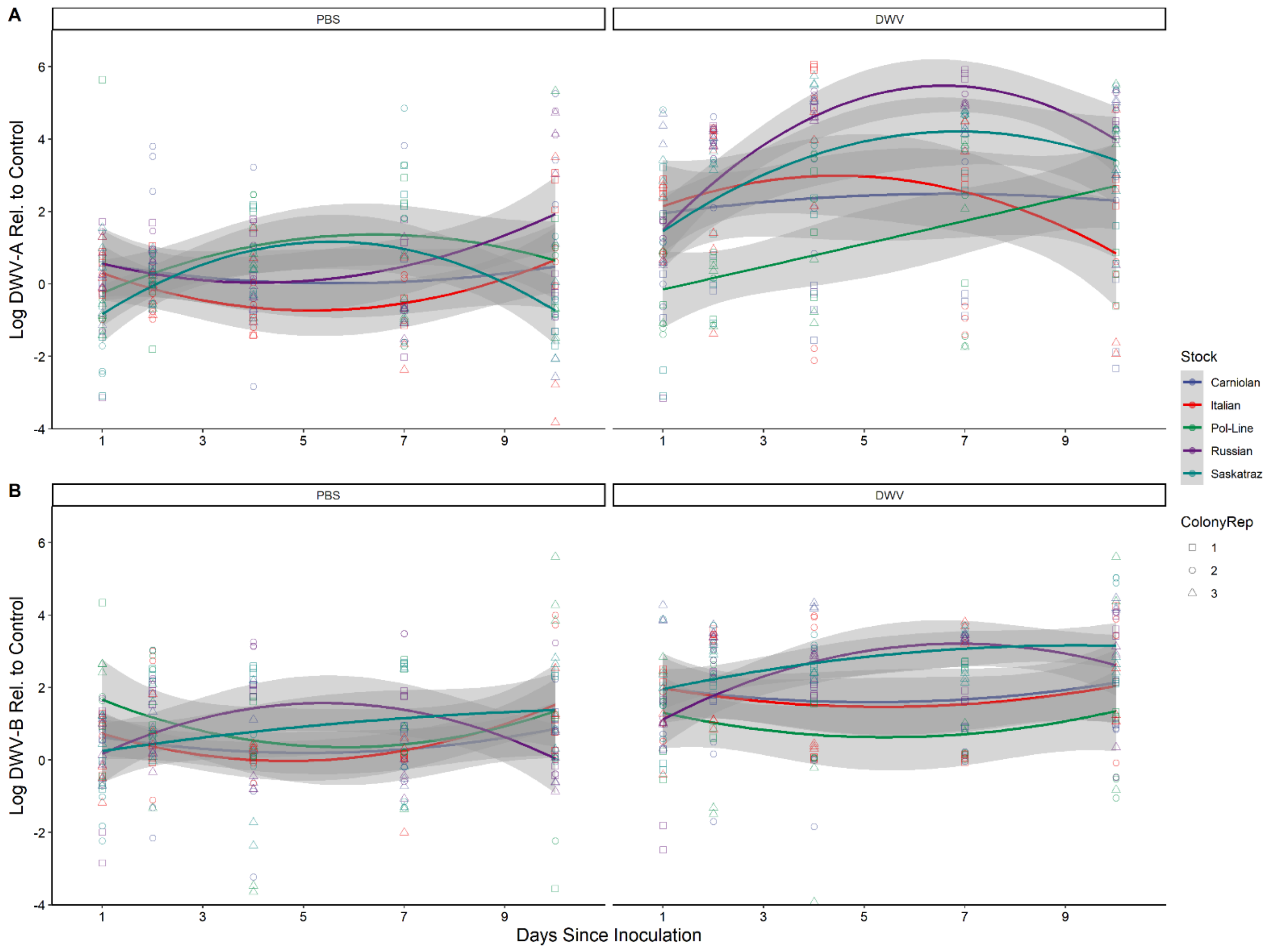

Supplementary Figure 4. DWV levels in *abdominal tissues* 1, 2, 4, 7, and 10 days after inoculation for the five genetic stocks for (A) Log DWV-A and (B) DWV-B levels. Gray areas represent the standard error for each line.

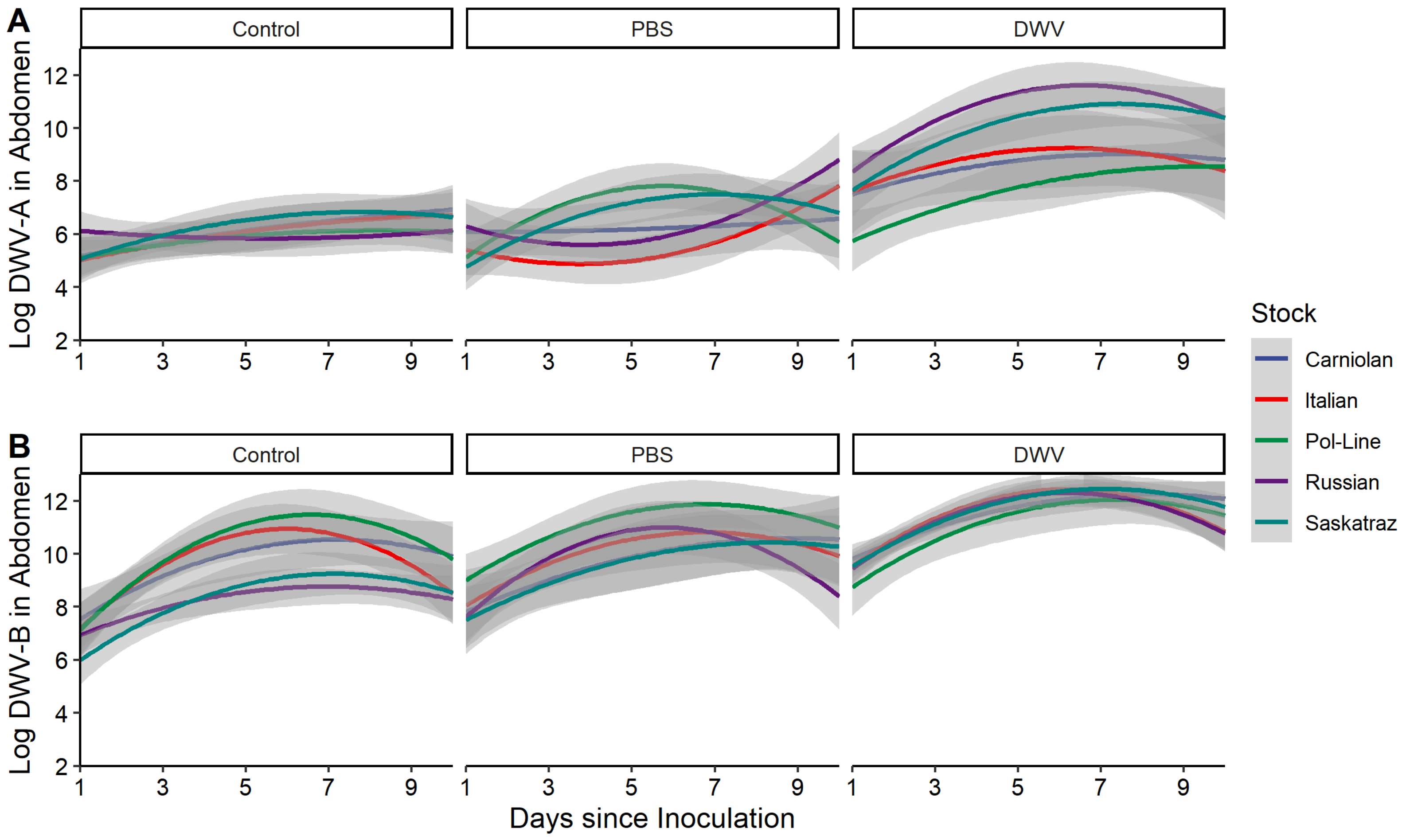

Supplementary Figure 5. DWV levels in *rear leg tissues* 1, 2, 4, 7, and 10 days after inoculation for the five genetic stocks for (A) Log DWV-A and (B) DWV-B levels. Gray areas represent the standard error for each line.

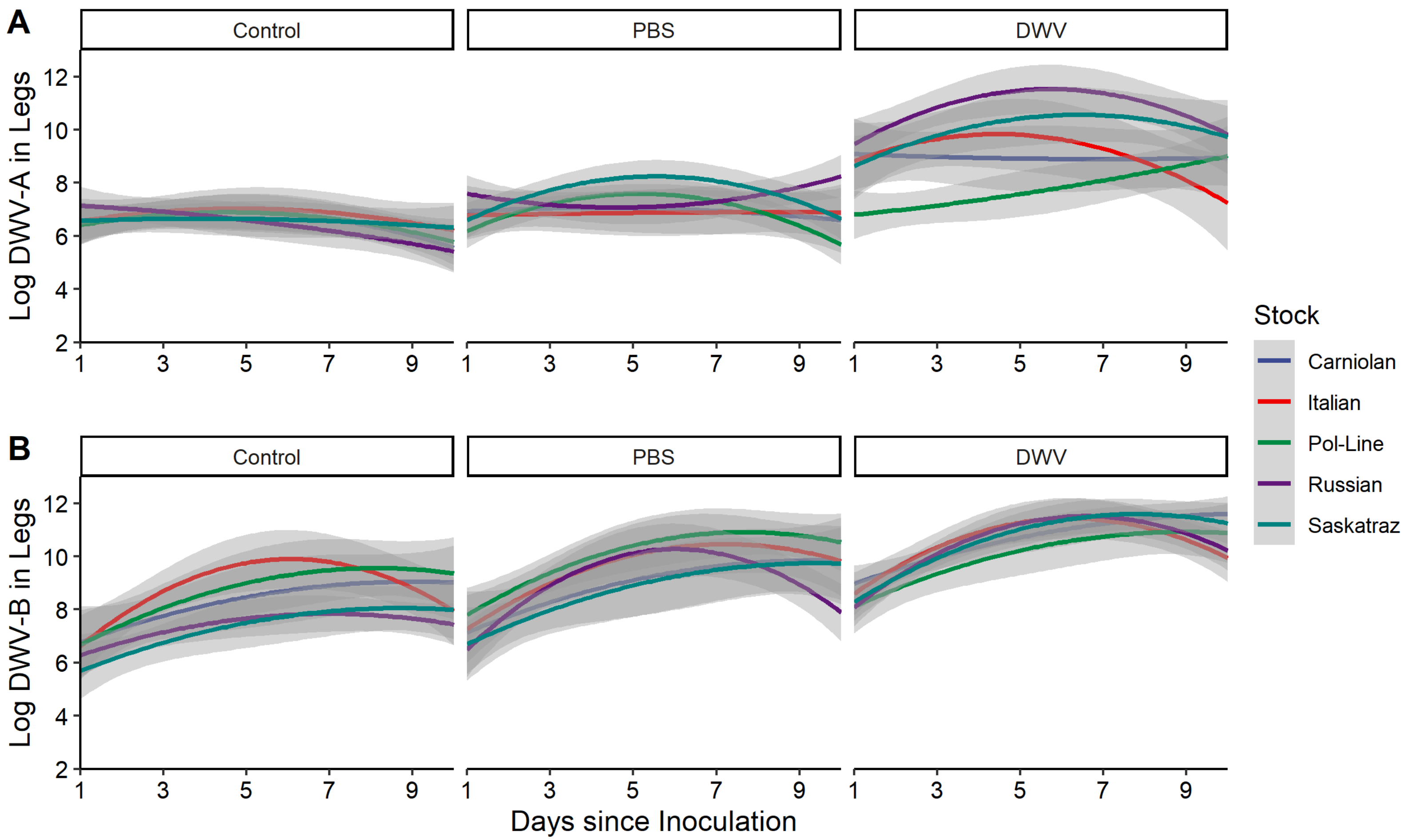

Supplementary Figure 6. DWV levels in *hypopharyngeal gland tissues* 1, 2, 4, 7, and 10 days after inoculation for the five genetic stocks for (A) Log DWV-A and (B) DWV-B levels. Gray areas represent the standard error for each line.

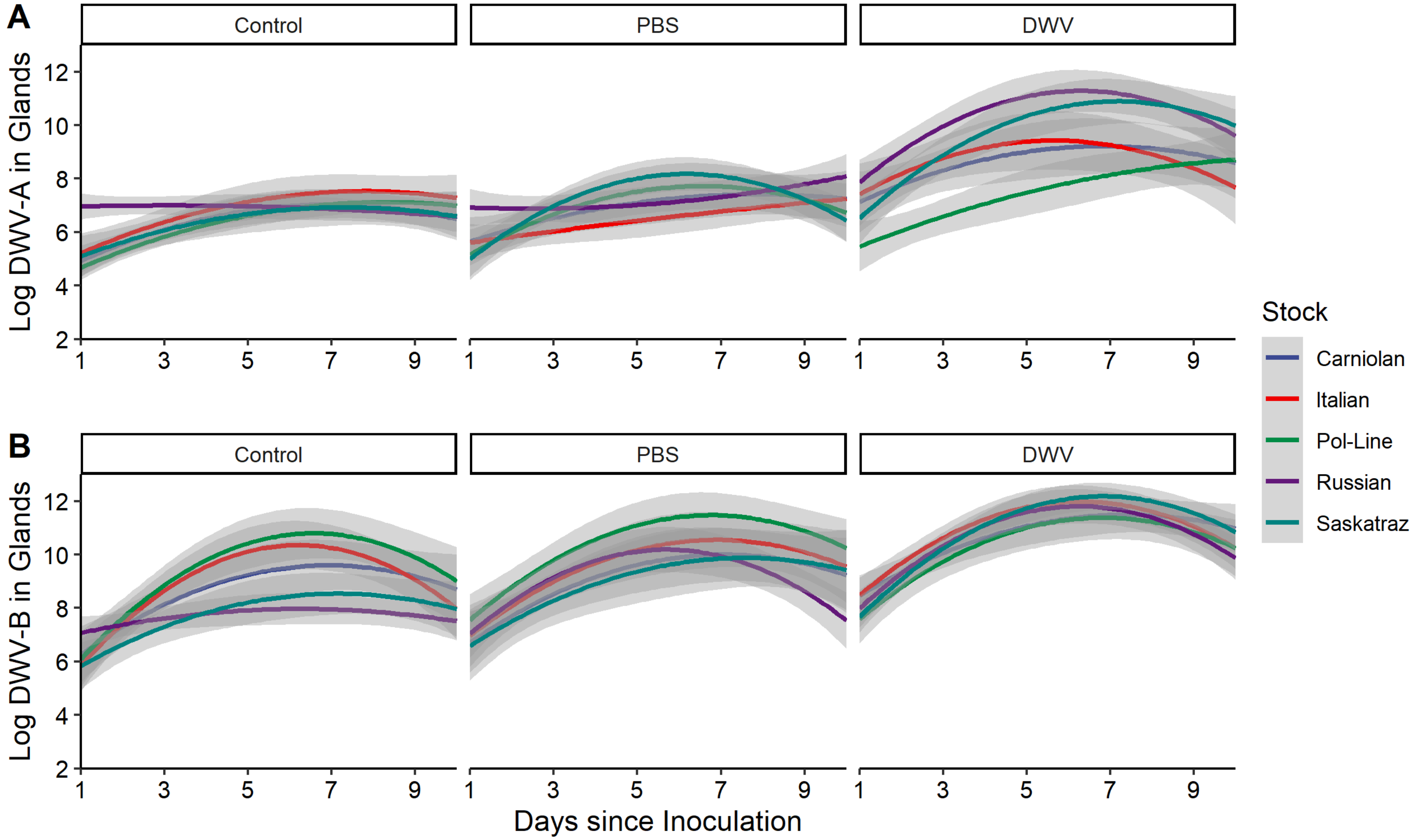

Supplement: Supplementary file 2 [file Data_Sheet_2.PDF]
